# Editorial: The Neurology of Global Lifestyle Change

**DOI:** 10.3389/fpubh.2020.614598

**Published:** 2020-11-19

**Authors:** Gerry Leisman, Ahmed A. Moustafa, Seema Biswas

**Affiliations:** ^1^Faculty of Health Sciences, University of Haifa, Haifa, Israel; ^2^Facultad ‘Manuel Fajardo' Instituto de Neurología y Neurocirugía, Neurofisiología Clínica, Universidad de Ciencias Médicas, Ciudad de la Habana, Cuba; ^3^School of Social Sciences and Psychology, The MARCS Institute for Brain, Behaviour and Development, Western Sydney University, Penrith, NSW, Australia; ^4^British Medical Journal of Case Reports, London, United Kingdom

**Keywords:** global health, neuroprotection, enrichment, cognition, movement, aging, child development

## Introduction

The nervous system allows organisms to interact with their environment. Effective living is contingent upon effective adaptive behavior (1). The developing child largely obtains the training necessary for nervous system development by environmental interaction (1, 2).

Nervous system dysfunction comprises a significant and growing global burden in treating disease and disorder producing loads on educational and governmental systems that support children with special needs as well as the health care needs of adults in the “Fourth Age.” Developmental disabilities, autism, dementias, stroke, traumatic brain injuries, PTSD, Parkinson's and Alzheimer's are significant influences governing mortality and morbidity worldwide. The means necessary to address the needs of individuals with disability remain disproportionately inadequate. An estimated 15% of the world's population lives with some form of disability (3). The shortage of rehabilitation facilities affects high-income countries as well as low, where individuals fare more poorly. The International Committee of the Red Cross Special Fund for the Disabled noted up to 80% of the world's population with disabilities are in countries with local health resources insufficient to meet rehabilitation needs (4).

Júlvez et al. (5) editorialized that in globally applying neurodevelopmental principles in health and educational systems we must simultaneously consider economic and cultural variability among populations. The epidemiology of lifespan neurodevelopment is pertinent for global governmental policy-making. Governments need to assess brain and behavior in a diverse global context.

Cognitive and neurological health is not simply a function of the existence of clinical neurological services but requires linkage to educational and human capital initiatives supporting and promoting optimized nervous system function. The Research Topic on the *Neurology of Global Lifestyle Chan*ge endeavors to highlight relatively simple means, easily implementable, to provide relatively inexpensive solutions to neurologically-based lifespan and cognitive-motor dysfunctions.

In high-income countries, there are 1–10 neurologists per 100,000 population (6). In contrast, public neurological and rehabilitation services are scarce or non-existent in low-income countries. The World Federation for Neurological Rehabilitation asserts the need for coordinated long-term investment in rehabilitation facilities, health-personnel training, minimum standards in neurorehabilitation, and community-based rehabilitation programs with research collaborations between clinicians and scientists. Investigators gathered in October 2014 in Barcelona to generate agreement in directives for epidemiological research to integrate knowledge the effects of environment on brain development, cognition, and policy (5).

A need exists for high-quality research into relatively scientifically-grounded interventions for effective cognitive and physical development as well as educational services based on sound principles of the neurophysiology of cognitive growth and sustainability. There is a paucity of literature regarding national and international strategies, for the management of those suffering from stroke or dementia, trauma and violence, and developmentally disabled (7, 8). Populations affected by disability fare worse in situations of poverty, disaster, and conflict (9). We need to continue our push for improvements in the global management of chronic neurological disease and disability, normal development and aging, neurodevelopmental interventions through early childhood education and in re-evaluating global health priorities.

The potential impact of research in neuroscience, disability and education on global health is considerable. A better understanding of factors responsible for brain development and behavior should significantly enhance our care of patients with congenital abnormalities. Eighty percent of children with cerebral palsy are born in low and middle-income countries (10). With under-resourced health care systems and families struggling with the basic necessities for living, research highlighting low-cost health interventions, effective health education programs, and strategies for improved chronic care are necessary. Relatively inexpensive health education programs within early childhood educational systems have the potential to influence not only health practices in adulthood but the optimization of cognitive efficiencies throughout the lifespan (11).

Better understanding of movement and motility disorders or the importance of movement itself in learning and in the prevention of cognitive decline could help populations affected by neurodegenerative conditions. Populations in India and China are facing massive global health challenges as the annual incidence of neurological disorders in Asia continues to grow faster than anywhere else (12).

We may be able mitigate some of the difficulties facing populations arriving at the “Fourth Age,” through lifestyle changes or health interventions earlier in life. Improvements in global neurological health must come with a better understanding of how to effect optimized human function based on how humans work, play, and perform activities of daily living.

A vigorous cognitive lifestyle relates to cerebrovascular disease reduction in males (12, 13). The literature reports that both men and women demonstrate neurotropic changes in prefrontal areas associated with cognitive lifestyle, consistent with a compensatory process (7, 8, 14). Complex cognitive endeavor throughout a lifespan may defend against dementia, with implications for populations in low-resource settings (13, 15, 16). Sufficient evidence exists to conclude that an integrated socially active lifestyle in the Fourth Age can offer neuroprotection for the dementias and for AD. The conclusions can be integrated simply into a unified theory of the nature of cognitive decline associated with advancing years in which different risk factors can be significantly reduced by employing neuroprotective methods.

Von Bismarck of Germany selected 65 as retirement age realizing that the average German worker would never reach that age. Fifty-five years later, the US passed a social security law when the average life expectancy was then 61.7 years (17). In Europe and elsewhere, retirement is now mandated for public employees, which by any standard is discriminatory and illegal, and of negative value for public health. Human resource managers often do not work with Social Security administrators or with Ministries of Health. This Research Topic adds to the evidence-base supporting active and productive lifestyles contributing to Public Health in the Fourth Age and how interventions early in life may impact health and cognitive status throughout the lifespan.

## Author Contributions

GL, AM, and SB equally shared in the writing of the editorial submitted here and in reviewing and organizing the Research Topic in all of its aspects. All authors contributed to the article and approved the submitted version.

## Conflict of Interest

The authors declare that the research was conducted in the absence of any commercial or financial relationships that could be construed as a potential conflict of interest.
